# Preoperative Enteral Nutritional Support in Patients Undergoing Hepatectomy for Hepatocellular Carcinoma: A Strengthening the Reporting of Observational Studies in Epidemiology Article

**DOI:** 10.1097/MD.0000000000002006

**Published:** 2015-11-20

**Authors:** Hui Yao, Xiaojie Bian, Liang Mao, Xuejian Zi, Xiaopeng Yan, Yudong Qiu

**Affiliations:** From the Department of Hepatopancreatobiliary Surgery, Drum Tower Hospital, Medical School of Nanjing University (HY, XB, LM, XY, YQ) and Department of Hepatopancreatobiliary Surgery, Nanjing Drum Tower Hospital Clinical College of Nanjing Medical University, Nanjing, China (XZ).

## Abstract

To compare the short-term outcomes between hepatocellular carcinoma (HCC) patients with and those without preoperative nutrition on the basis of postoperative enteral nutrition.

HCC patients with postoperative enteral nutrition who underwent liver resection between February 2010 and December 2014 in Nanjing Drum Tower Hospital were considered for the study: 43 patients with and 36 patients without preoperative nutrition. Primary endpoint was the incidence of overall complications. Secondary endpoints were infectious and major complications.

In the preoperative enteral nutrition group, shorter length of postoperative hospital stay (10.5 ± 2.7 versus 13.7 ± 6.3 days, *P* = 0.007), less exogenous albumin infusion (10.2 ± 22.4 versus 47.8 ± 97.7 g, *P* = 0.030), earlier first exhaust time (2.7 ± 0.8 versus 3.0 ± 0.9 days, *P* = 0.043), and first defection time (3.5 ± 0.9 versus 4.4 ± 1.4 days, *P* = 0.001) were observed. No significant differences were observed in the incidence of overall complications (32.6% versus 52.8%, *P* = 0.070), infectious complications (7.0% versus 8.3%, *P* = 1), and major complications (14.0% versus 11.1%, *P* = 0.969) between the preoperative enteral nutrition and control group.

Preoperative enteral nutrition could improve short-term outcomes of HCC patients via accelerating the recovery of gastrointestinal function and shortening the length of postoperative hospital stay.

## INTRODUCTION

Hepatocellular carcinoma (HCC) is the most common type of liver cancer with high mortality rate worldwide.^1^ It is the sixth most common cancer and the third most common cause of death from cancer worldwide.^2^ In eastern Asia, the etiology of liver cancer incidence is mainly because of chronic infection with hepatitis B viruses.^3^ Nowadays, the classification of patients and the most appropriate personalized therapy are based on the Barcelona Clinic Liver Cancer system. According to it, HCC in patients with preserved liver function can be treated curatively by hepatectomy.^4,5^ Although morbidity and mortality after liver resection for HCC have been improved by the refinement of surgical techniques and perioperative management in the last decades, the rate of postoperative complications remains high, with an incidence of 28% to 47%.^6–10^ Therefore, there is a great need for strategies to reduce the incidence of postoperative complications.

With the recent development of dietetics, perioperative nutrition has been not only a tool to supply calorie and nitrogen support, but also a therapeutic strategy aimed at enhancing the immune system and increasing resistance to complications.^11^ The European Society for Parenteral and Enteral Nutrition recommended patients with hepatectomy to initiate enteral nutrition within 12 to 24 hours postoperatively, which can minimize the incidence of perioperative particularly infectious complications.^12^ Early postoperative enteral nutrition has been confirmed to reduce catabolism, decrease stress reaction, lessen postoperative complications, and accelerate recovery in patients with partial hepatectomy for primary liver cancer.^13–15^

The value of preoperative enteral nutritional (pEN) support in patients with hepatectomy, however, remains questionable.^12^ The recent studies failed to confirm the effect of pEN on postoperative complications after liver resection.^16,17^ Therefore, the authors investigated the effect of pEN on short-term outcomes in patients who underwent hepatectomy for HCC.

## METHODS

### Patients

In the retrospective cohort study, the clinical data of 79 HCC patients with postoperative enteral nutrition who underwent curative liver resection in Nanjing Drum Tower Hospital between February 2010 and December 2014 were retrieved and collected. All liver resection specimens had tumor-free resection margins. The diagnosis of HCC was confirmed by histology or cytology. Patients without enteral nutrition after surgery, patients who had undergone treatment, such as transarterial chemoembolization and ablation therapy before hepatectomy, and patients who had a palliative liver resection, those with a histologically positive resection margin were excluded from the study. The following information were collected by the authors: age, sex, hepatitis, cirrhosis, pathology, histology, tumor-node-metastasis (TNM) classification, Child–Pugh grade, model for end-stage liver disease score, body mass index, type of liver resection (major ≥3 versus minor <3 Couinaud segments), tumor number, tumor size, anatomic hepatectomy, portal triad clamping time, operation time, estimated blood loss, blood transfusion, perioperative nutrition support, serum total bilirubin on day 7 after hepatectomy, exogenous albumin infusion, drainage, postoperative complications, mortality, intensive care unit (ICU) admission, first exhaust time, first defecation time, length of hospital stay, postoperative hospital stay, postoperative duration to enteral feeding, enteral nutrition-related side effects (diarrhea, bloating, vomiting, constipation, electrolyte imbalance, and glucose metabolism disorders), and postoperative levels of serum Na^+^, K^+^, and glucose.

Patients were divided into 2 groups: pEN group and control group. Patients in pEN group were given nutritional support before surgery with oral-impact, enteral nutritional suspension (total protein-medium chain triglycerides [TP-MCT], 1 kcal/mL), 500 to 1000 mL per day for 3 consecutive days. Nutrition was added to normal diet. Patients in control group had only normal diet without enteral nutritional support before surgery. All patients in the 2 groups were given enteral nutrition after surgery. Preoperative enteral nutritional support was introduced in the authors’ department for liver surgery in 2010. Patient's compliance with nutrition support was verified by the nurse.

All patients gave informed written consent for surgery. The retrospective study did not require ethics approvals because of the monocentric study design and data analysis without data transmission.

### Surgical Technique

In brief, after an upper abdominal midline incision or right subcostal incision with upward midline extension, liver parenchymal transection was performed by a cavitron ultrasonic aspirator. Hemostasis was achieved by argon beam coagulation, saline-linked monopolar electric cautery, and fine suturing. Intermittent portal triad clamping was applied during liver transection only if excessive bleeding was encountered. Intraoperative ultrasonography was performed routinely to determine tumor location, detect any major vascular infiltration, and mark the plane of liver transection. The solution of methylene blue was injected into the cystic duct or hepatic duct after transection to detect the bile leakage. Albumin was given when the level of serum albumin was below 30 g/L. If necessary, patients were monitored in the ICU for at least 24 hours after surgery. According to the guidelines, antibiotics were given perioperatively.^18^

### Postoperative Management

There was no significant modification except for the nutritional support in the perioperative management during the study period. All patients in the 2 groups were given nutritional support by nasojejunal tube after surgery with enteral nutritional suspension (TP-MCT, 1 kcal/mL), 500 to 1000 mL per day for at least 5 consecutive days. Patients were discharged when all the following criteria were met: oral analgesics could control pain well, solid diet could be tolerated, intravenous rehydration was not needed, bilirubin levels were normal or nearly normal, and smooth bowel movements and activity levels returned to preoperative levels.^19,20^

### Definitions

Postoperative complications were defined and categorized by the Clavien–Dindo classification.^21^ Ascites was defined as fluid draining from the drainage tube exceeding 300 mL/d for more than 3 days. Reactive pleural effusion could be determined by x-ray and ultrasound examinations. Wound infection was defined as swelling and exudation at the surgical site with or without dehiscence.

Atelectasis or pulmonary infection could be diagnosed by the symptoms and signs with the assistance of abnormalities on chest x-ray films. Intraperitoneal hemorrhage from the residual liver's surface or the diaphragm might be indicated by persistent bloody drainage. Gastrointestinal tract bleeding could be manifested by brown or bloody drainage, melena, hematemesis, and be diagnosed by endoscopic examination.

Bile leak was defined as the drainage of exceeding 50 mL of bile from the surgical drain or from drainage of an abdominal collection.^22^ Liver failure was defined as concomitant hyperbilirubinemia on or after the fifth day postoperatively, along with prolonged international normalized ratio.^23^

### Endpoints

The primary endpoint was the incidence of overall complications. Secondary endpoints were the incidence of infectious and major complications.

### Statistical Analysis

All statistical analyses were performed with Predictive Analytics SoftWare statistics version 18.0 (SPSS Inc., Chicago, IL). Continuous variables were presented as mean with standard deviation, and compared using the Student *t* test. Categorical variables were compared with the χ^2^ test, Fisher exact test, or nonparametric Mann–Whitney *U* test, as appropriate. All *P* values <0.05 were regarded to be statistically significant.

## RESULTS

### Baseline Characteristics

The retrospective cohort study consisted of 79 HCC patients with postoperative enteral nutrition who underwent curative liver resection in our Department from February 2010 to December 2014. Patients were divided into the 2 groups: pEN group (n = 43) with pEN and control group (n = 36) without pEN. The baseline characteristics of patients were well matched between the 2 groups and outlined in Table 1. The pEN group was comparable with the control group with regards to age, sex, body mass index, hepatitis, cirrhosis, Child–Pugh grade, model for end-stage liver disease score, TNM-stage, and microvascular invasion (all *P* > 0.05). Besides, the intraoperative data of patients between the pEN group and the control group were not significantly different with regards to tumor number, tumor size, type of hepatic resection, anatomic hepatectomy, portal triad clamping time, estimated blood loss, intraoperative blood transfusion and operation time (Table 2; all *P* > 0.05).

**TABLE 1:**
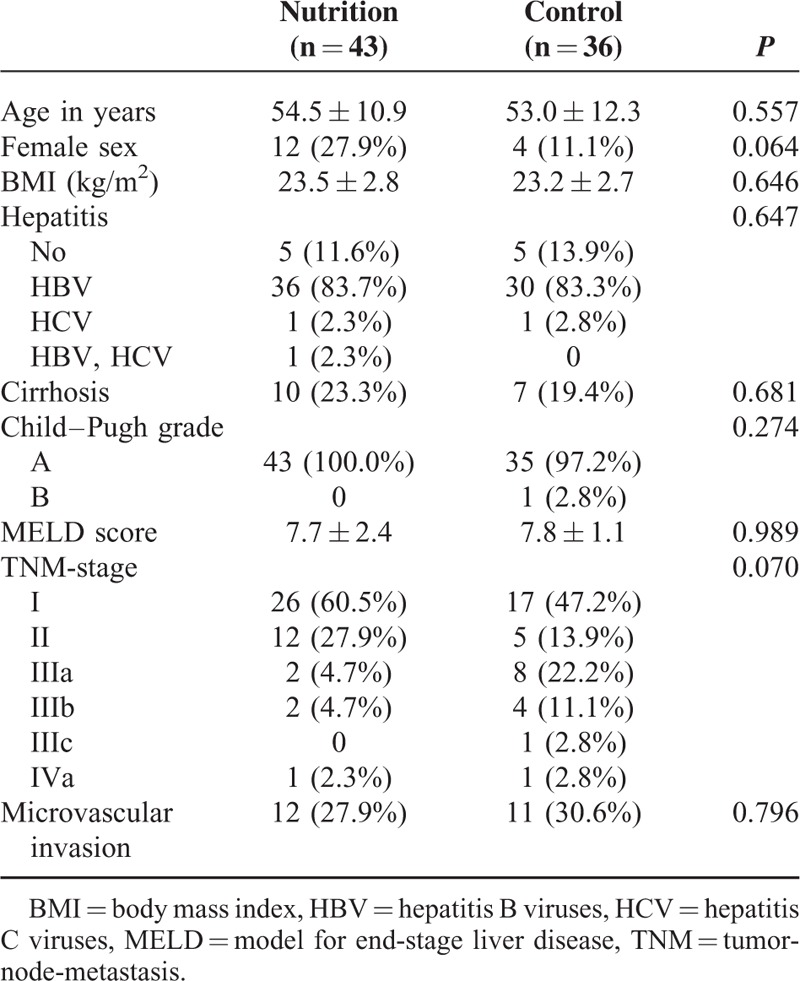
Baseline Characteristics of Patients in the Preoperative Enteral Nutrition Group and in the Control Group

**TABLE 2:**
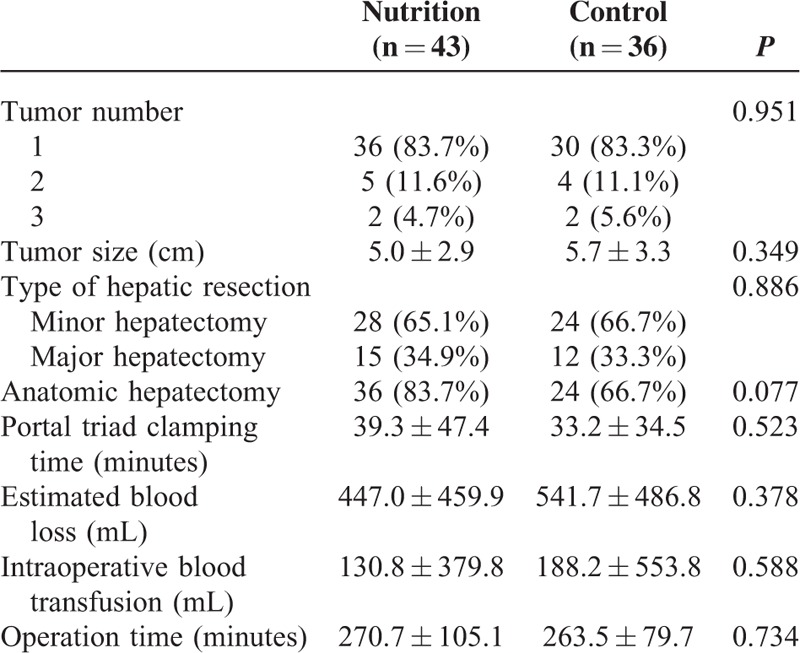
Intraoperative Data of Patients in the Preoperative Enteral Nutrition Group and in the Control Group

### Postoperative Outcomes

Postoperative complications of patients between the 2 groups were shown in Table 3. Although the number of patients with postoperative morbidity in the pEN group was lower than that in the control group, the difference was not significant (32.6% versus 52.8%, *P* = 0.070). There was no significant difference in the complication grade between the 2 groups (*P* = 0.117). No significant difference was found in the incidence of major complications between the pEN group and the control group (14.0% versus 11.1%, *P* = 0.969). Also, there was no significant difference in the number of patients with infectious complications between the pEN group and the control group (7.0% versus 8.3%, *P* = 1). The most common complication was ascites, representing 16.3% and 41.7% of the pEN group and the control group, respectively (*P* = 0.012). Postoperative recovery of patients between the 2 groups was shown in Table 4. There was no significant difference in the serum total bilirubin on day 7 after hepatectomy between the pEN group and the control group (17.6 ± 8.3 versus 20.9 ± 8.6 umol/L, *P* = 0.089). There was no significant difference in the number of patients with postoperative ICU admission between the pEN group and the control group (34.9% versus 38.9%, *P* = 0.713). Also, there was no significant difference in the length of hospital stay between the pEN group and the control group (18.5 ± 4.6 versus 20.8 ± 7.6 days, *P* = 0.108). Patients in the pEN group, however, had significantly shorter postoperative hospital stay than those in the control group (10.5 ± 2.7 versus 13.7 ± 6.3 days, *P* = 0.007). Besides, patients in the pEN group had significantly earlier first exhaust time (2.7 ± 0.8 versus 3.0 ± 0.9 days, *P* = 0.043) and first defection time (3.5 ± 0.9 versus 4.4 ± 1.4 days, *P* = 0.001) than those in the control group. Patients in the pEN group had significantly less postoperative exogenous albumin infusion than those in the control group (10.2 ± 22.4 versus 47.8 ± 97.7 g, *P* = 0.030). No significant difference was found in the postoperative duration to enteral feeding between the pEN group and the control group (1.8 ± 0.9 versus 2.1 ± 0.7 days, *P* = 0.112). The enteral nutrition-related side effects in the pEN group were less than those in the control group (25.6% versus 41.7%), but this difference was not statistically significant (*P* = 0.130). The most common side effect was diarrhea, representing 25.6% and 41.7% of the pEN group and the control group, respectively (*P* = 0.214). Postoperative levels of serum Na^+^, K^+^, and glucose between the pEN group and the control group were not significantly different (Table 5; all *P* > 0.05).

**TABLE 3:**
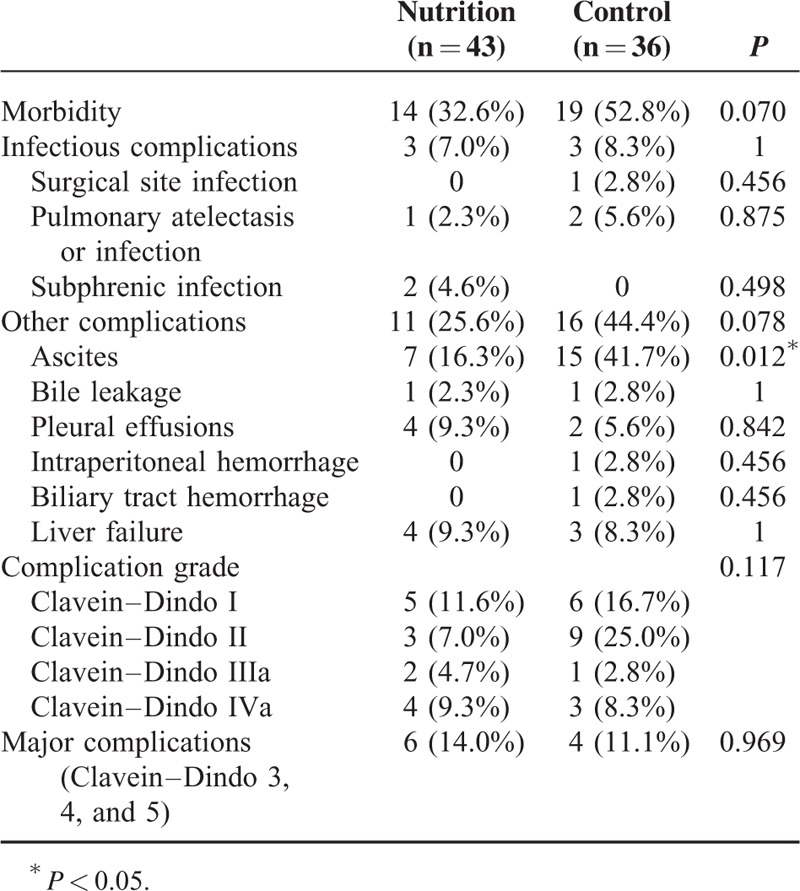
Postoperative Complications of Patients in the Preoperative Enteral Nutrition Group and in the Control Group

**TABLE 4:**
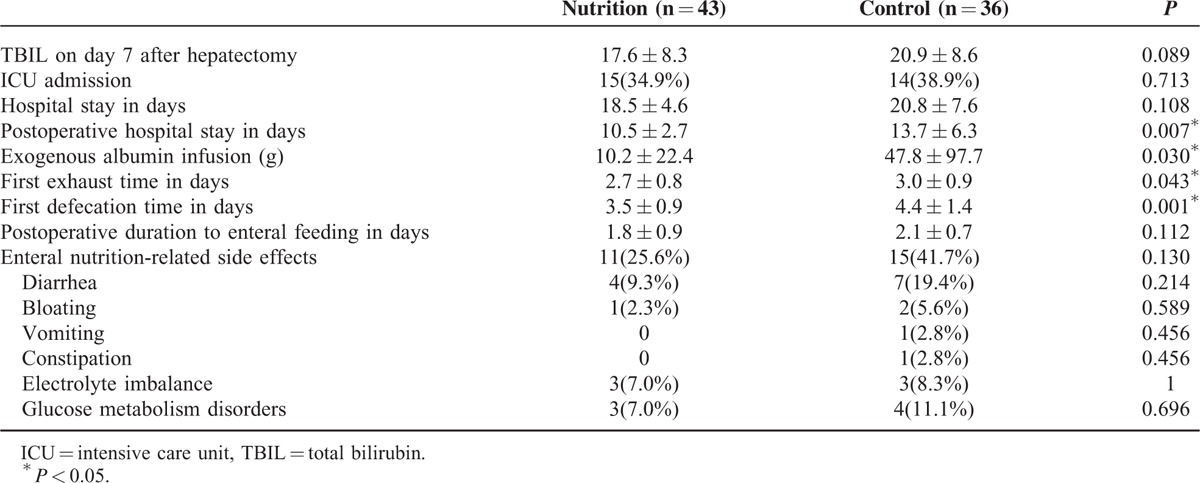
Postoperative Recovery of Patients in the Preoperative Enteral Nutrition Group and in the Control Group

**TABLE 5:**
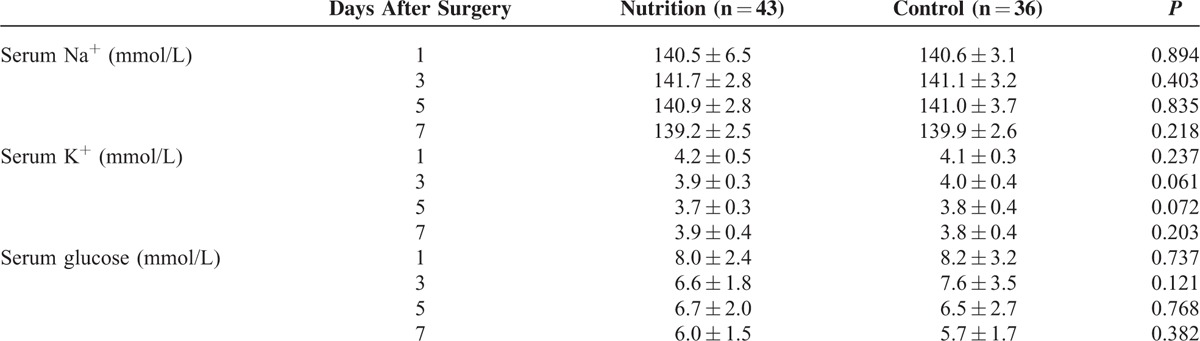
Postoperative Levels of Serum Na^+^, K^+^, and Glucose in the Preoperative Enteral Nutrition Group and in the Control Group

## DISCUSSION

The aim of the current study was to compare the short-term outcomes after liver resections between HCC patients with postoperative enteral nutrition who received and those who did not receive pEN. Although the current study did not prove an impact of pEN on postoperative complications after liver resection, pEN could still improve postoperative recovery via accelerating the recovery of gastrointestinal function and shortening the length of postoperative hospital stay.

In recent years, with the development of surgical techniques and perioperative management, particularly the fast-track surgery, the outcomes of patients with liver cancer after liver resections have been improved.^24–27^ And nutritional support plays a central role in the management of these patients, owing to the possibility of wide functional parenchyma ablation.^28–30^ The European Society for Parenteral and Enteral Nutrition recommended early postoperative enteral nutrition to the patients with hepatectomy.^12^ The value of pEN in liver resection, however, still remains questionable. It has been proved that pEN may reduce the length of stay, postoperative overall and infectious complications rate in pancreatic and gastrointestinal surgery.^31–35^ Patients with liver cancer undergoing liver resection, however, were not included in these trials.

Mikagi et al analyzed in a randomized trial the effect of pEN versus no nutrition in liver resection and proved a decreased postoperative inflammatory response. But the incidence of postoperative complications and length of postoperative hospital stay were not significantly different between the pEN group versus the control group.^16^ A review by Koretz et al,^36^ however, investigated pEN support for surgery in liver disease. And they did not find any differences with regard to complications in these trials.

Similarly, a propensity score matched case-control analysis performed by Zacharias, et al investigated the value of preoperative nutritional support in liver resection between patients with pEN and without preoperative nutritional support. They did not demonstrate an impact of pEN on postoperative complications after minor liver resection, which may be because of the absence of postoperative enteral nutrition.^17^

The current study, which did not demonstrate the effect of pEN on postoperative complications in patients with HCC undergoing liver resection, seems to confirm these trials. But, we proved that pEN could improve the postoperative recovery with low exogenous albumin infusion, early recovery of gastrointestinal function, and reduced length of postoperative hospital stay. In recent studies on enhanced recovery after hepatectomy, postoperative hospital stay based on functional recovery had been considered as the most important indicator.^37,38^ All patients in the current study were discharged in accordance with the above criteria. Therefore, the authors considered that reduced postoperative hospital stay in pEN group was because of faster recovery of gastrointestinal function and earlier tolerance of solid food, which was in line with the concept of fast-track surgery. Besides, the authors observed that pEN might reduce the side effects of postoperative enteral nutrition, such as bloating, diarrhea, nausea, vomiting, constipation, electrolyte imbalance, and glucose metabolism disorders. Therefore, the authors assumed that pEN could improve the inadaptability of postoperative enteral nutrition, and further trials were needed to confirm the hypothesis.

### Limitation

In the retrospective cohort study, the absence of randomization remains the major limitation. Although the baseline characteristics of patients between the preoperative enteral group and the control group were well matched, patients in an early TNM-stage were more frequently observed in pEN group. On the contrary, anatomic hepatectomy was 66.7% in pEN group versus 83.7% in control group. It has been shown, that anatomic hepatectomy could improve outcomes, particularly in the present of microvascular invasion.^39^

Besides, the current study was performed by a single center, and some patients were excluded, thus the number of patients was small. The “negative” results of pEN observed in the present may be explained by type II error.

The further limitation in the current study may be the absence of preoperative nutritional risk assessment, which can be performed by nutritional risk assessment tool, such as Nutritional Risk Screening 2002, Subjective Global Assessment, Malnutrition Universal Screening Tool, and Nutritional Risk Index. It has been shown, that higher nutritional risk score was related with adverse effects, which may lead to poor prognosis.^40,41^

Although these limitations, the current study could be regarded as a real situation of enteral nutrition for patients with HCC who underwent liver resection in the authors’ institution. The authors expected the pEN could reduce the incidence of postoperative complications on the basis of the postoperative enteral nutrition. The authors, however, observed reduced rate of side effects of postoperative enteral nutrition, and further study would be needed.

## CONCLUSIONS

The current study did not confirm an impact of pEN on postoperative complications after liver resection, but pEN could still improve postoperative recovery via accelerating recovery of gastrointestinal function and shortening the length of postoperative hospital stay.
